# Clinical research coordinators’ role in knowledge translation activities in rehabilitation: a mixed methods study

**DOI:** 10.1186/s12913-023-09027-0

**Published:** 2023-02-07

**Authors:** P. Kengne Talla, C. Robillard, S. Ahmed, A. Guindon, C. Houtekier, A. Thomas

**Affiliations:** 1 School of Physical and Occupational Therapy, McGill University, Montreal, Canada; 2 Integrated University Health and Social Services Centre for West-Central Montreal (CIUSSS du Centre-Ouest-de-L’Île-de-Montréal), Montreal, Canada; 3 Integrated University Health and Social Services Centre for South-Central Montreal (Institut Universitaire Sur La Réadaptation en Déficience Physique de Montréal, CIUSSS du Centre-Sud-de-L’Île-de-Montréal), Montreal, Canada; 4 Centre for Interdisciplinary Research in Rehabilitation of Greater Montreal, Montreal, Canada; 5 Department of Sexology, Université du Québec À Montréal (UQAM), Montreal, Canada; 6 Integrated Health and Social Services Centre of Montérégie-Centre (CISSS de La Montérégie-Centre), Institut Nazareth Et Louis-Braille, Montreal, Canada; 7 Institute of Health Sciences Education, McGill University, Montreal, Canada; 8 Integrated Health and Social Services Centre of Laval, CISSS de Laval, Montreal, Canada

**Keywords:** Knowledge translation, Rehabilitation, Determinants, Clinical research coordinator, Implementation strategy

## Abstract

**Background:**

Clinical research coordinators (CRCs) facilitate the interaction between researchers and knowledge users in rehabilitation centres to promote and sustain evidence-informed practices. Despite their presence in rehabilitation settings in Quebec for over 20 years, little is known about their profiles and knowledge translation (KT) activities nor how they can best enact their role. This study explored CRCs’ roles and perspectives on the barriers, enablers, and strategies for improving KT activities in rehabilitation settings.

**Methods:**

We conducted a multi-centre, participatory sequential mixed methods study. In the descriptive quantitative phase, we collected data via an online survey to determine CRCs’ role in research and KT. In the subsequent qualitative phase, we conducted an in-person focus group to elicit CRCs’ perspectives regarding factors influencing their work in KT, and potential solutions for overcoming these challenges. We used a descriptive and an inductive content analysis approach for the data analysis. The data synthesis was inspired by the Promoting Action on Research Implementation in Health Services framework.

**Results:**

All nine CRCs from five partner health regions of a large rehabilitation research centre agreed to participate in the study. The data suggest that CRCs are like knowledge brokers and boundary spanners. As information managers, linkage agents and facilitators, CRCs play a pivot role in diffusion, dissemination, synthesis and tailoring of knowledge to improve evidence informed practices and quality of care in rehabilitation. The factors influencing CRCs’ KT activities are mostly linked to the context such as the receptivity of the organization as well as the lack of time and resources, and limited understanding of their roles by stakeholders. Two main suggestions made to enhance CRCs’ contribution to KT activities include the harmonisation of expectations between the large research centre and their partner health regions, and better promotion of their role to clinical and research teams.

**Conclusions:**

This study provides valuable insights into the scope of CRCs’ role. The results shed light on the challenges that they face and potential solutions to overcome them. The knowledge generated in this study can be used to implement this role with similar duties in rehabilitation settings or other health care domains.

**Supplementary Information:**

The online version contains supplementary material available at 10.1186/s12913-023-09027-0.

## Background

Over the last two decades, studies have demonstrated that evidence-informed practices (EIP) are essential to high-value care and efficiency of healthcare systems [1, 2]. EIP consists of combining the best evidence based on research and patient care data with patient preferences and values, and the health professional’s expertise to make a clinical decision [3]. Knowledge translation (KT) aims to promote the use of EIP in health care to reduce the gap between the latest evidence and clinical practices and to ensure that research findings inform clinical decision-making [4]. The process of EIP can reduce costs and avoid wasting healthcare resources [5, 6]. However, there is a need for greater clarity regarding the factors that influence EIP at the patient, the provider, the organization’s physical and social environment, and policy or societal levels [7, 8], as well as the optimal implementation strategies to support any EIP [9].

There is a growing evidence base on various strategies (e.g., such as passive, active, tailored, discrete, and multifaceted interventions) [10–18] aiming to enhance the adoption, implementation, and sustainability of innovations in health [19–22]. This literature suggests that multifaceted and interactive strategies that involve various stakeholders are most promising in achieving EIP [23]. Among those, knowledge brokering (KB) is a multidimensional implementation strategy with the potential to i) facilitate interaction among researchers and knowledge users; ii) promote mutual understanding of goals related to the integration of evidence into practice and policy-related decisions [23, 24]; iii) help build new partnerships [25]; and iv) build stakeholder capacity in implementation of research evidence in practice for a sustainable change [26, 27]. The nature, complexity and breadth of knowledge brokering depends on the local needs [28] and may be influenced by tensions between the type of knowledge (e.g. tacit, scientific), aspects of knowledge brokering (e.g. linkage, exchange) and by the KB’s intermediary position as a catalyst of change [27, 29]. In brief, KBs can improve the organizational readiness for EIP based on the premise that interpersonal contact among KB and the stakeholders improves the likelihood of behavioral change associated with the use or adoption of new knowledge [30]. Research shows that KBs have used multiple interventions such as workshops, seminars and public meetings to increase knowledge and evidence-based practices among knowledge users [31].

The role of knowledge brokering in healthcare settings is often held by managers, clinicians, or other professionals within the clinical environment or externally (e.g., in a research centre or a private structure as a consultant) [29, 30]. There are variations in the terms used to describe a person who takes on a KB role. For instance, a recent study on KBs in rehabilitation reported that this role is predominantly filled by clinicians on a part-time basis, and that various terms are used to refer to KBs including capacity builder, linkage agent, facilitator, evaluator and information manager [26, 32, 33]. In the biomedical field more broadly, a comparable role is termed clinical research coordinator (CRC) [34] which includes duties related to the recruitment of research participants and project management, legal and regulatory compliance, budget preparation, database management [35, 36], and ensuring adherence to the best clinical practices [37, 38]. While CRCs in this study have some common tasks (e.g., project coordination, or budget management) as those in the biomedical field, their roles and the source of employment and salary are often particular. CRCs in rehabilitation work closely with the clinical program (i.e., clinicians, managers, patients) and all researchers in a research centre. They act as a bridge between researchers from the research centre and clinical teams in the health regions by developing a mutual understanding of goals, facilitating the communication between these people from two different cultures, supporting them to collaborate to find issues and ultimately to promote the integration of the best available evidence into clinical decision-making. They are not hired by principal investigators as a research support personnel to coordinate a research study in comparison to CRCs in the biomedical field working in close proximity to the principal investigator who leads the clinical research study [38]. CRCs’ in the biomedical are accountable to a principal investigator with a salary dependent on his grants [39]. Thus, CRCs and KBs can promote EIP via their broad and comprehensive understanding of the organization.

Despite the potential support that CRCs could bring to individuals and organizations, little is known about their profiles, their roles/responsibilities, activities, and the extent of their involvement in KT activities. Filling this gap and developing an empirical account of CRCs’ roles in KT activities is important in helping us (to) understand the relevance of these roles in the creation of new knowledge and to promote EIP. The overarching aim of this study was to explore CRCs’ roles within their setting and perspectives on the barriers, enablers, and strategies for improving KT activities in rehabilitation practice.

## Methods

### Study design and setting

We adopted a multi-centre, participatory sequential explanatory (quantitative–qualitative) mixed methodological approach [40, 41]. Congruent with a participatory approach, most participants were involved in all phases of the study [42]. The data from the quantitative phase were used to develop the interview guide for the subsequent qualitative phase. Data collection occurred between June 2018 and March 2020.

The study involved five partner health regions of a large rehabilitation research centre which is the Centre for Interdisciplinary Research in Rehabilitation of Greater Montreal (CRIR) in Quebec, Canada. These health regions each have their own context, territory, and client population. Despite these diverse characteristics, they share the common goal of improving the well-being and participation of individuals living with disabilities. These partner health regions are composed of several clinical site programs including a rehabilitation centre composed of clinicians from many professions (e.g., occupational therapists, physiotherapists and social workers).

The CRIR is a unique, interdisciplinary and intersectoral research centre, and is associated with six health regions and four universities in Quebec promoting excellence and innovative research in rehabilitation [43]. In this position, CRIR builds a research culture within its partners’ health regions and their clinical programs in rehabilitation. The linkages between the clinical teams and the researchers within the CRIR is a large part of the roles and responsibilities of a CRC. CRCs are employees of these health regions in the same way that clinical teams are. They work across multiple rehabilitation centers in partner health regions of CRIR. In each rehabilitation center, CRCs report to a formal supervisory whose department depends on each local context.

#### Participants

The number of CRCs ranges from one to four per health region. Nine CRCs across the five partner health regions participated in our study. The CRIR has played a major role in establishing the CRCs at the rehabilitation centers. They are highly trained professionals who provide coordinated and structured activities for clinicians, managers and patients. They collaborate with CRIR researchers to meet the needs of people with physical, hearing and visual disabilities.

#### Quantitative phase

##### Data collection

We collected data using a web-based questionnaire mounted on the Lime-Survey platform. The research team developed questions based on the scientific literature and their expertise in KT. The self-administered questionnaire (see Additional file 1) included 37 questions (both multiple choice and open-ended questions) divided into three sections: i) sociodemographic information (*n* = 24 questions); 2) work activities with specific stakeholders e.g., managers, clinicians, and their KT process (*n* = 10 questions) on a scale from always, usually, sometimes, rarely to never; or weekly, biweekly, monthly, annually and never; or always, often, sometimes, rarely and never; and 3) confidence and satisfaction with their role in these KT activities (*n* = 3 questions) on a 5 point Likert -scale from strongly agree to strongly disagree. The remaining questions were multiple choice and participants were asked to choose the answer from several options. Respondents could add any additional information in a comment section at the end of the questionnaire. Approximately 25 min was necessary to complete the questionnaire. Two former CRCs who no longer worked in the participating health regions piloted the questionnaire for clarity, comprehensibility, relevance, and length. We made minor modifications following their comments. A research assistant sent an email inviting all CRCs to participate in the study. Two reminders were sent (at weeks 2 and 4).

##### Data analysis

We used descriptive statistics (percentages and frequencies) to summarize participants’ characteristics and content analysis [44, 45] to analyze the free-text answers. We used the results from the quantitative analysis to develop the interview guide for the subsequent phase. The qualitative results helped to interpret and support part of the quantitative results. These results were integrated during the data analysis and interpretation.

#### Qualitative phase

##### Design and data collection

For the qualitative component, we used a descriptive design [46, 47] and conducted one focus group discussion (FGD) with the CRCs [48]. We developed the focus group interview guide based on findings from the survey and a review of relevant literature in KT. The main sections in the interview guide were: i) their roles in KT within their health institutions with examples of successful and failed KT activities; ii) the barriers and enablers towards KT activities and; iii) what they would need as a solution or a mechanism/strategy to fully carry out their roles as CRCs in KT.

Three research team members reviewed and then revised the guide for clarity. During the focus group, we asked participants to consider the definition of KT from the Canadian Institutes of Health Research [49] to promote common ground for the discussion. The interview guide is available upon request. The FGD was moderated by two individuals with experience in this approach. It lasted 2.5 h and was audio-recorded and transcribed verbatim.

##### Data analysis

Two members of the research team independently performed an inductive content analysis [44, 45] using NVivo software v.12 [50]. Data analysis involved three phases: 1) familiarization with the data; 2) grouping of quotes into emerging categories and 3) the organization of the data within a categorization matrix deducted from the project’s objectives and questions. Discrepancies were resolved through discussions with the rest of the research team. Trustworthiness of the data was addressed through triangulation of data collection methods (focus groups and surveys) [51, 52], and the use of multiple coders. We addressed credibility [53] by 1) cross-checking audio-files and transcripts by the facilitators of focus groups, 2) ongoing discussion in small groups with some members of the research team about the coding and the results, and 3) peer-review of preliminary results with all CRCs to ensure their perspectives were clearly represented in the analysis. All quotes in the article are free translations. The members of the research team included CRCs and researchers. They have worked closely for many years and have a detailed understanding of their local contexts as the clinical programs and the CRIR. This common knowledge was considered useful to elicit their diverse and multiple viewpoints on their roles within their rehabilitation centers. Once all content of the focus group was summarized into emerging themes, each theme was then regrouped into categories reflecting first the research objectives (barriers and enablers to performing KT activities). Second, we used the Promoting Action on Research Implementation in Health Services (PARIHS) framework [54] to synthesize our data on the factors influencing the KT activities from CRCs’ perspectives. The PARIHS framework is a determinant framework used to understand better the practical aspects of moving evidence into practice and explore barriers and enablers influencing implementation outcomes. However, it is often used as an organizing framework for data analysis [55]. The PARIHS is considered one of the most commonly used determinant frameworks to study the role of context in implementation [56] and in the research to understand knowledge brokering activities [57].

According to this framework, successful implementation depends on the nature of the evidence, the context in which the change will be implemented and the mechanisms by which the change is facilitated. The context refers to the environment or setting in which the proposed change is to be implemented [54] or can differentiate between the immediate local level, the wider organizational level and external health system level [58]. According to PARIHS, the context is subdivided into three core elements: an understanding of the prevailing culture, the leadership roles and the organization's approach to the measurement. Other key aspects of context include: i) the relevance of the innovation to the organization, ii) the organizational fit of the innovation to organizational structures and procedures, iii) adequate resources for implementation, and iv) the use of implementation strategies. The facilitation refers to the type of support needed to help people change their attitudes, habits, skills and ways of thinking and working. The following dimensions are identified within the facilitation role namely personal characteristics; clearly defined roles; and styles of working. To this study, we considered only two components of the PARIHS framework in the data synthesis, namely the context and the facilitation because the nature of evidence was beyond the scope of this study.

## Results

### Sociodemographic data

All nine CRCs participated in both stages of the study. Table 1 shows that eight of the nine CRCs were female and 36 years old or older. Four respondents were members of their professional associations and held a doctorate or had completed a post-doctoral fellowship (44%). Seven CRCs are accountable to the research and academic affairs directorate, held a permanent position and worked at least 4 days/week (mean = 28 h and SD = 7) with more than 60% of their time devoted to the clinical research coordinations. A few CRCs indicated an academic affiliation (22%). The average pay rate was CAN $45 per hour, excluding benefits. A job description was available and accessible for six of the nine participants. For some of them, their fields of education were not relevant in their role as CRCs (56%).

**Table 1:** Characteristics of participants

Variables	Frequency (*n* = 9)
Age group	
Under 35 years old	1
36–50 years old	4
Over 50 years old	4
Gender	
Male	1
Female	8
Highest academic level	
Master's degree	5
Doctorate degree	4
Member of a professional licence body	
Yes	4
No	5
Academic affiliation	
Yes	2
No	7
University master program	
Biomedical Sciences and Physiotherapy	1
Community health	1
Epidemiology/Biostatistics	1
Information Sciences	1
Psychology	1
Sexology	1
Social work	1
Social and Cultural Anthropology	1
Speech Language/Pathology	1
Areas of University doctorate program	
Biomedical Sciences and Physiotherapy	1
Clinical psychology	1
Community Health	1
Social and cultural anthropology	1
Number of hours per week in current position	
35 h/week	2
32 h/week	3
28 h/week	1
21 h/week	2
17.5 h/week	1
Proportion of hours spent on CRC position	
100%	4
Under 100%	4
Question not understood	1
Perception about the relevance of own education for CRCs’ work	
Yes	5
No	4
Permanent position	
Yes	7
No	2
Hourly rate for CRCs	
Less than $45	3
More than $45	5
NR	1
CRCs’ management department (where CRCs report to)	
Clinical rehabilitation directorate	1
Multidisciplinary services directorate	1
Research and academic affairs directorate	7
Availability of a formal job description	
Yes	6
No	3

#### CRCs’ perceptions about their research and KT activities

We used data from the questionnaire to create this table. As seen in Table 2, the time that CRCs invest in the research and KT activities varies. For example, the most common bi-weekly activities were: i) facilitating the review process for research projects in their healthcare institution (institutional suitability and feasibility); ii) managing the research projects by maintaining a database of ongoing projects; iii) improving internal communication with other CRCs or individuals in similar positions, as well as with individuals outside their healthcare institutions, and iv) optimising the identification of the strategies to recruit participants for research projects.

**Table 2:** Perception on research and KT activities from CRCs’ perspectives

List of functions in research	Frequency (*n* = 9)
Maintain a database of ongoing projects in the institution	8
Facilitate the review process for research projects that involve your institution (institutional suitability)	7
Facilitate the identification of strategies to recruit participants for research projects	7
Communicating with people within or outside your organization, such community members, patients, caregivers, other health care professionals, government personnel, or others	6
Support activities that promote exchange between researchers and stakeholders, including clinical teams or managers	5
Work with stakeholders including local clinical teams or managers to identify clinical needs or questions that could be addressed by research projects	5
Find evidence-based practices through activities such as setting up automatic journal alerts or reading articles	5
Provide support for the management of research projects (e.g. support for ethics applications, project monitoring)	5
Communication with other CRCs or people in similar positions, collaborate with people outside the institution	5
Organize scientific conferences to present research results to stakeholders including clinical teams or managers	4

Most CRCs mentioned that their roles are not well known to most clinicians, managers, researchers, students, and research assistants. All CRCs felt competent and respected by others and were autonomous in their roles. Eight of the nine CRCs felt valued and confident in their work and expressed satisfaction in their work. However, eight of the nine CRCs agreed that they need support or training to optimise their roles (Table 3).

**Table 3:** CRCs’ perceptions about the confidence and the satisfaction towards their roles (*n* = 9)

Variables	*Strongly agree*	*Agree*	*Neutral*	*Disagree*	*Strongly disagree*
Clear description of my position	1	1	2	5	0
Clarity of my duties	2	3	2	2	0
Clarity of my duties for my immediate manager	2	3	2	2	0
Clarity of my duties to stakeholders	0	2	1	6	0
Clarity of my activities for other managers with whom I work	0	4	0	5	0
Clarity of my activities for researchers, students, research assistants	0	4	0	5	0
Clarity of my activities for the other people I work with	1	4	1	3	0
I am called upon for the right reasons	0	3	3	3	0
I am autonomous in my work	5	4	0	0	0
I feel valued in my work	3	5	1	0	0
I feel respected in my work	5	4	0	0	0
I feel confident in my work	2	6	1	0	0
I feel competent in my work	2	7	0	0	0
I have enough time to accomplish my goals in my work	0	1	2	6	0
I would like more support in my role as a CRC	3	5	00	1	0
I would like to have more training to fulfill my role as a CRC	2	6	1	0	0
I enjoy my work	3	5	1	0	0

#### CRCs’ KT activities, barriers, enablers and strategies

##### CRCs’ KT activities

These results were derived from data analysis of both quantitative and qualitative phases. CRCs support KT via activities that can be grouped into three main categories (Table 4).

**Table 4:** Facilitation/CRCs’ roles in knowledge translation activities

Level	Strategies/mechanisms	Subcategories	Representative quotations
**Facilitation**	Role as translator, mediator, transmission belt, gatekeeper, knowledge broker (e.g. a translator for the clinical teams of the functioning of the academic world and the clinical realities; an intermediary to ensure a common language between researchers and managers (mediator role); a transmission belt of knowledge between the key actors, i.e., clinical knowledge and experiential knowledge)	Information manager: Knowledge diffusion and dissemination (e.g. conferences during the lunchtime, websites, community of practice	"I think our role so far, because of lack of resources, is mainly diffusion—it's passive. There is no real integration of knowledge" (# CRC 1) "…We have a calendar that is more structured, it's every three months, I think? Anyway, which is quarterly and with lots of activities that are varied, that reach out to diverse audiences qualitatively and probably quantitatively…. researchers, students who are the carriers of these activities of scientific animation, but it remains that the target public also, it is very varied that it is students, researchers, partners, users…Even if we organize conferences during the lunch time, there's always an opening for other activities." (# CRC 2) "It's something that we have a lot of time to do. It's pretty easy to organize a conference, we don't have much to do …." (# CRC 3) "… Helping them write the application, prepare the poster, submit the abstract to the symposium. Just thinking about the project, itself or the presentation, structuring the ideas" (# CRC 1)
	Role as translator, mediator, transmission belt, gatekeeper, knowledge broker (e.g. a translator for the clinical teams of the functioning of the academic world and the clinical realities; an intermediary to ensure a common language between researchers and managers (mediator role); a transmission belt of knowledge between the key actors, i.e., clinical knowledge and experiential knowledge)	Information manager: Assessing and synthetizing information (e.g. **s**ummary or synthesis of knowledge in clear, simple and concise language targeting clinical teams disseminated electronically, web-page)	."Writing of a newsletter (10 times a year); Feeding the research Web pages; Writing of the annual report on research activities; Support to a community of practice of clinicians Support for the development of research projects Support for publication by clinical teams (posters, presentations) Responding to ministerial mandates.". (#CRCs 1,2,3,4,5,6,7,8,9) "The transfer of knowledge begins the moment a project is presented to a manager, so it takes a certain amount of accompaniment so that everyone can speak the same language, and I have often found that the manager did not fully understand the project, so I had to organize a meeting with the researcher, or at least arrange for them to speak together. So that's what I think is central to my role." (# CRC 6) "The academic affairs department had a meeting with the researchers (…) to find out what the main obstacle was…. And then one of the things that came up was the common language, a lack of common language. It's really, not only that they have two different realities or two different rhythms, clinical and research, they don't speak the same language at all." (# CRC 1)
	Role as translator, mediator, transmission belt, gatekeeper, knowledge broker (e.g. a translator for the clinical teams of the functioning of the academic world and the clinical realities; an intermediary to ensure a common language between researchers and managers (mediator role); a transmission belt of knowledge between the key actors, i.e., clinical knowledge and experiential knowledge)	Information manager: Providing resources (e.g. scanning of relevant information such as articles, evidence-based papers, or useful websites; marketing of research articles for the implications at their local context; Assisting clinical teams in the writing of a paper or the creation of their materials such as posters or oral presentations at local, national, and international	"We have a marketing role. Projects like that that are less sexy, or …. it doesn't fit as well, and then you must sell it, so we have a certain loyalty to our researchers, a loyalty to the institution means that (…) that's one of the difficulties we have too." (# CRC 1)
		Facilitator: Centralization of requests from research teams targeting the clinical teams; Coordination of projects; Organization of KT activities coordinated with and for the clinical program, e.g., presentation of ongoing projects to clinical teams; Organization of informal meetings	"The best triangle is the research knowledge, the organizational knowledge and the experiential knowledge. My role is to circulate as much of this knowledge as possible…I know that clinicians are interested in research, but research is interested in clinicians. (# CRC 5)
		Linkage agent and capacity builder: Sensitize the research team to the clinical context in which the project is taking place; Encourage to having a member of the clinical team of the targeted program on the research team and increasing the matching and the connections; Raise awareness’ research team on knowledge transfer strategies that can be carried out in synergy with clinical needs and realities)	"We are actually called CRC, but we could be called knowledge brokers too" (# CRC 9) "The linkage … is between the two, but we are very useful for that, to make…any project to be possible" (# CRC 8) "Actually, I also see our role as an intermediary, as someone who also comes…" (# CRC 2) "We are in the middle, we are translators of both languages. Translation of everyone's realities, translation of everyone's objectives, interests, motivations… we are there." (# CRC 1)

The first category of KT activities in which CRCs are involved is knowledge dissemination, synthetizing and tailoring the resources to their stakeholders where they have a role of information manager. This includes the diffusion and dissemination of knowledge through e-newsletters, and web pages, organising scientific conferences for diverse audiences, as well as finding new evidence and resources that may be of interest to the clinical teams. CRCs customize their KT activities to the target audience, including patients, clinicians, managers, researchers, students. However, they are less involved in active practice change.

For instance, one CRC said: “*We are strongly encouraged to realize KT activities of various forms … but it remains that the [target audience] can change to include students, researchers, partners, patients…” (# CRC 2).*

From another participant: “I think our role so far, because of lack of resources, is mainly diffusion – it’s passive. There is no real integration of knowledge." (# CRC 1).

The second category of KT activities is the reinforcement of a scientific culture among clinical teams through: i) organizing regular meetings with managers to increase their awareness of the research at their rehabilitation center, as well as its relevance to the clinical practice; ii) co-developing research projects, and supporting clinical projects in collaboration with researchers; iii) following up on projects within the clinical program and offering regular presentations of projects in progress; iv) consulting clinical teams in the identification of evidence-based tools to promote scientific literacy. In these functions, they play a role of facilitator and linkage agent.

For instance, one CRC said: *"Given that there is a lot of turnover among managers, it is interesting to meet with them to re-explain [the project], to make a presentation on the university mission, [to learn about] the expectations from managers in the clinical programs, [to visit] clinical teams and managers….to find out how we could communicate with them, what would be the best communication channel? How can we reach them easily? How can we transfer knowledge to them?…*” *(# CRC 2).*

The third category of KT activities is the development of opportunities for collaboration, communication and co-learning among the research and clinical teams. CRCs are employed in settings that are ripe with opportunities for building and sustaining relationships between stakeholders by facilitating mutual understanding of each group’s needs and expectations. In these roles, CRCs support them in reflexive learnings by helping to identify their needs. Here, CRCs have a role of a facilitator and a capacity builder. This was exemplified in the following quotes:

"The best triangle is the research knowledge, the organizational knowledge and the experiential knowledge. My role is to circulate as much of this knowledge as possible. So yes, I know that clinicians are interested in research, but research is interested in clinicians.” (# CRC 5).

"The knowledge transfer begins once a project is presented to a manager. This takes a certain amount of guidance to ensure everyone speaks the same language, and I have often found that the manager did not fully understand the project, so I had to organize a meeting with the researcher, or at least arrange for them to speak together. So that's what I think is central to my role.” (# CRC 6).

CRCs used different terms to describe their role of bringing together the research and clinical environments, including mediators, facilitators, translators, or intermediaries.

"We are in the middle; we are translators of both languages. Translating each other's realities, translating each other's goals, interests, motivations… we're there." (# CRC 1).

"The linkage we are talking about is between researchers and clinical teams, but we are very useful for that, to make this kind of interaction facilitator so that any project is possible" (# CRC 8).

"We have a marketing role. Projects like that are less sexy, or …. it doesn't fit as well, and then you must sell it….” (# CRC 1)

#### CRCs’ perspectives on their needs and strategies to enhance their role

Table 5 provides a summary of facilitation strategies to enhance the CRCs’ role including: i) a set of skills and attributes required to perform an effective facilitation role such as personal, interpersonal and synthesis skills. CRCs mentioned that it takes certain personal attributes to enact this role, such as strong communication skills, listener, humility, project management abilities, and knowledge synthesis skills.

**Table 5:** Facilitation to improve CRCs’ knowledge translation activities

Level	Strategies/mechanisms	Subcategories	Representative quotations
**Facilitation**	Facilitator	Having a particular profile with personal skills, deprecation, communication and synthesis skills (e.g. to be able to work closely with clinical teams and managers; to be strategic mediator)	"Then one thing we forgot to mention is us as a professional, as a person. You know, it takes a certain profile of person to be the middle of all that. …And with certain skills, personal abilities, all of that, not just anyone could…, would be good in that role. I think that around the table we do a good job." (# CRC 1) "Communication skills, communication synthesis I think is the common trait.." (# CRC 7) "Actually, in addition to the strategic skills, there are project manager skills because every single thing that we undertake often is a project. And we're a mix between project manager and firefighter because sometimes (laughs) but it's true, some days it's both… and secretarial luxury (laughs)." (# CRC 9)
		Style of facilitation: Feeling as competent, autonomous, valued, respected, confident and enjoy her/his work	No quote/from quantitative data analysis
		Time to increase the integration and appropriation of evidence; Need of human resources;	."We are trying more and more to develop other types of activities even if it is difficult and it takes time… we didn't have enough time to do it." (# CRC 3) "The support of having administrative agents, the more external support you have, the easier it is to work. So that's what I said (laughs) about the facilitators. Have a project management training …yes that had been extremely helpful." (# CRC 8)
	Type of needed support	Training tailored our needs such as project management, using social medias in KT; Budget to ensure clinical involvement in research and KT; Alignment & marketing of CRCs’ role; Clear action plan with the targets and expectations	"Support for practice changes, I don't think we have a role in that either at this point. Is that part of our role? Maybe not so much, but I think there's still a willingness to use part of our role to see (…) how we can bring evidence into the programs. Because we don't have that many other resources." (# CRC 3) "You're talking about resources, that there's maybe a piece that comes from the clinical programs themselves …. There's a piece of resources that would come from them." (# CRC 7) "So I think there will definitely be things to do in the future to try to raise awareness (…) Yeah that's it [marketing the profession, suggested by another participant] … I think it helps but it's really not everyone who knows it." (# CRC 3) "You were talking about an action plan, um, that's more at the institutional level, but let's say that if large research centre were to set out wishes or expectations in terms of results to be obtained in an x environment in terms of knowledge transfer, and that this information be shared by the management to which we report (…) and that the managers be the bearers of this as well. It would help a lot to align things because otherwise (…) Let it be at the institutional level but also at the large research centre level and let it be shared so that the information is common to all sites So after that, how it's articulated could vary from setting to setting but there would at least be explicit expectations…" (# CRC 9) ..we all have our personal and site specificities, but I think that there would still be something to do potentially…" (# CRC 3)

For example, one CRC said: *"In addition to the strategic skills, we have project management skills because everything we do is often in the form of a project. We are a mix between a project manager and a firefighter because sometimes (laughs) but it's true, some days it's both." (#CRC 9).*

"Then one thing we forgot to mention is us as a professional, as a person. You know, it takes a certain profile of person to be the middle of all that. …And with certain skills, personal abilities, all of that, not just anyone could-, would be good in that role. I think that around the table we do a good job" (# CRC 1).

ii) a better connection and harmonization with the clinical and the research centres’ directorates; iii) a marketing approach to increase awareness of the CRC’s role; iv) an action plan developed in collaboration with the scientific directors and clinical managers, that includes clear indicators, targets, expectations of CRCs’ KT activities as well as the evaluation process, v) time and budget to improve the clinicians’ release for the KT activities and the integration of research results beyond the dissemination; and vi) training in project management, using social media as a KT tool and accessing various digital tools beyond diffusion strategies such as videoconferences. That is exemplified from following quotes:

"You were talking about an action plan, that's more at the institutional level, but let's say that (…) Let it be at the institutional level but also at the [the large research centre] level and let it be shared so that the information is common to all health regions. So, after that, how it's articulated could vary from setting to setting but there would at least be explicit expectations…" (# CRC 9).

"So I think there will definitely be things to do in the future to try to raise awareness (…) Yeah that's it [marketing the profession, suggested by another participant]..,I think it helps but it's really not everyone who knows it." (# CRC 3).

### Barriers to KT activities

Participants highlighted the importance of contextual factors when planning and implementing KT activities. As illustrated in Table 6, CRCs described several barriers at the organisational level related to their role in KT: (i) lack of administrative support; ii) limited availability of clinicians and managers who have to consider performance expectations; ii) limited budget for help with clerical activities; (iii) limited availability of researchers to develop effective KT strategies; (iv) overlap of CRCs’ responsibilities with the individuals in their institutions who are involved in practice change, and (v) limited access to up-to-date material resources or digital tools for KT.

**Table 6:** Factors influencing CRCs’ knowledge translation activities

Level	Factors	Subcategories	Representative quotations
**Context/Inner setting**	**Organisational barriers**	Lack of time (for instance, CRC have not enough time to accompany practices changes)	"…The change can be very, very long and then the researcher doesn't necessarily have the time to do it. We certainly don't have the time to do that and then neither do the clinical teams…It takes a learning curve." (# CRC 1) "One issue I've been dealing with recently. A researcher proposed in her research project to have a training component, at least a workshop, at the end (…) [She] wanted to integrate this into a program meeting for clinicians who would have participated in the research, and then we came up against the manager who said, 'well, no, I don't have the time to integrate this into my program meetings'. So, it's not that the researchers are unwilling. Sometimes it's even how it fits in, when it's super relevant but they just don't have time to fit a 2-h training into a meeting that lasts an hour sometimes." (# CRC 6)
		Lack of human and informational resources (for instance infrastructure, access to technology, lack of clinical staff for KT activities)	"Neither CRC nor large research centre are structured to package or disseminate validated practice or assessment tools developed by researchers." (# CRC 2) "The situation that I have however in the programs it's not about the money necessarily. You know, like Participant 3 says, there is interest, there is motivation, we are supported by the managers, but there is 50% of the staff that is missing. You know, the clinical teams, they can't give more than not for recruitment, for knowledge transfer, for presentations, they just can't, they don't have the resources anymore." (# CRC 1)
		Lack of clarity on CRCs' mandates i. Researchers do not know the role of CRC, which is perceived as an administrator and an obstacle, it is not really clear for them the brokering role ii. Great variability in CRCs’ roles according to the reality of health regions and large research centre iii. Confusion on their roles and no common vision of CRCs’ roles between large research centre, clinicians	"I was seen as an interposing person and I explained that … is asking for this, it is the clinical environment., but before, I was going to see the occupational therapists, I was going to see the physiotherapists, I was asking them for this, I was asking them for that". And now I said no, the requests must be centralized to the managers and it goes through a CRC…But, the reality is really that it's not a requirement of our boss (…) It's really the clinical settings." (# CRC 4) "Anything called knowledge mobilization, going into programs and integrating and fostering practice change, I speak for [me], there, I don't see it as being in my role definition." (# CRC 7) "The closer we get to appropriation and then to changing practices, the more our mandate becomes blurred precisely because of the links with the DMS, the DSI, the DSP and the CIUSSS…" (# CRC 8) "I also find that researchers within large research centre believe that we are people who block things and then don't do much or understand anything. But I think that they don't really know what our role is (…) which may also create frustrations because they have the impression that our role is just to block projects or to protect programs too much… No one has a common vision for CRCs [The large research centre] has its vision, we have our own vision for our role, the researchers have another, and the clinicians have another one." (≠ CRC 3)
		Duality between the managers’ involvement in research and their challenges to respond to the ministerial requirements about performance indicators	"Then within management, people always have good will, have lots of ideas, …. but they also have their own constraints that come down from the department or whatever so it's not quite realistic to integrate that into their reality…" (# CRC 9) "We were talking about organization that has changed in the last few years, all of that, I'm not sure that it's returned for example in the research culture, this so important reality of streamlining time and context, the degree of freedom of clinicians that has reduced, I'm not sure that all researchers have realized that…" (# CRC 4) "Then we often have several realities in silos because large research centre, it's not that well known. In any case, in my field, among clinicians, if I asked people what large research centre is, I'm sure I'd get all sorts of answers, including huh? What is it? and I can't blame them. It's normal, it's not their day-to-day." (# CRC 9)
		Cultural differences between the rehabilitation institutions and the large research centre (e.g. lack of common language, different agenda)	
	**Organisational enablers**	Fertile ground for a culture of research and scientific thinking among university graduates where they need to support the excellence required by accreditation process and encourage the evidence-based practices (e.g. Access to other institutes and expertise within healthcare institutions; alignment with the strategic direction; to be a team with more than one CRC to exchange ideas and maximize expertise (on same site), organization of work is facilitating.)	"I've worked in several other places, there is an interest from the clinical settings. There's a research culture. … people come out of university, they've been exposed to a high level of scientific thinking and they want to keep that, so that, for me, is really in terms of knowledge transfer, it's a hyper fertile ground. That doesn't mean that everything is facilitated around that but that, I think, is a great, great lever." (# CRC 4) "It is facilitating to have strategic directions with targets and indicators which give an intentionality to KT." (# CRC 2) "I find that we really have the support of management of rehabilitation and in research. They are motivated… they spend time in meetings with us to see how we can improve the activities we do in research to listen to us, to hear what we have to say and then I think that the fact that they are motivated… it's a factor really, at least for me I find it a factor that is super helpful in what we do." (# CRC 3)
		Supportive management from clinical programs the large research centre (e.g. Motivated managers who listen; managers with a high interest and a scientific literacy) Availability of resources (e.g. budget for clinicians’ release and coverage of their time to participate in research or develop posters; administrative support)	
**Context/Outer setting**	**Organisational enablers**	Specific structure of the large research centre (e.g. Consortium of several healthcare institutions with the easy access to researchers and knowledge from elsewhere; networking with other CRCs)	"She [the KT resource person at large research centre] was pushing us to try to promote certain projects for example, every project and new initiative should present… It's true because the large research centre gives money, there is an obligation to present at the end." (# CRC 8) "I think it's also a plus to hear a little bit about everyone's reality. We have the same challenges, I would say, we don't have the same organization, but to see the successes from elsewhere, I find that very, very inspiring. Sometimes, the projects that you say, I say to myself -ah how could that inspire me? Maybe not that project but something else, I find that really fun." (# CRC 4) "Our re-networking through large research centre to say that we have access to researchers and knowledge that is collected in other centers, it's a multiplier then…" (# CRC 7) "There is something that I find that is a great facilitator and it's not always done is when the researcher plans for knowledge transfer money in their application for funds, that they plan that at the end, they will be able to give trainings, to package a guide of something…when they do that, it's a great facilitator." (# CRC 4) "Another positive point that I wanted to bring was the proximity of some researchers physically on site. That is a great advantage. …people have the desire to go and hear about (researcher) because they see her, they know that he/she is on the site. I think that there is an advantage in that people really take the time to come to the site, that their students come, I think that this is a lever for knowledge transfer." (# CRC 4)

From a CRC: *"The situation that I have however in the programs it's not about money necessarily. You know, there is an interest and motivation, we are supported by the managers, but there is 50% of the staff that is missing. Clinical teams can't give more for recruitment, for knowledge transfer, for presentations, they don't have the resources anymore ." (| CRC 1).*

Another one of the CRCs mentioned*: "When we get to the appropriation and change in practice, our mandate becomes unclear precisely because of the links with other departments at the level of the institution". (# CRC 8).*

CRCs noticed some confusion in the research and clinical environments regarding their mandates. The perception of CRCs’ responsibilities is not common between the managers of the large research centre, researchers and clinicians. From one CRC: *"No one has a common vision for CRCs. [The large research centre] has its vision, we have our own vision for our role, the researchers have another, and the clinicians have another one. Nobody has a common vision of what a CRC is. " (# CRC 3).*

### Facilitators for KT activities

Table 6 contains the details on the contextual (inner and outer settings) factors which can facilitate CRCs’ KT activities.

The context is related to the organisation in which the evidence is introduced into practice. Among the contextual level facilitators, CRCs mentioned: i) the support from managers such as the approval of the releasing time for clinicians to participate in the research activities; ii) the fertile research ground, which refers to the organizational climate and the cultural dimensions where there is the proximity between the clinical teams and the researchers, having many CRCs in the same institution, and the requirements for the accreditation; and iii) the availability of resources likely the administrative support for the clerical activities, colleagues’ expertise in KT, the anticipated budget for clinicians’ involvement in KT activities in grants and early discussion with the clinical teams and researchers on the type of KT activities.

From one participant: *"I find that we really have the support of rehabilitation and research managers. They are motivated…they spend time in meetings with us to see how we can improve the activities we do in research, to listen to us, to hear what we have to say and then I think that the fact that they are motivated… it's a factor really, at least for me I find, a factor that is super helpful in what we do." (# CRC 3).*

Other participants outline: *"The support of having administrative agents, …. the more external support you have, the easier it is to work. So that's what I said (laughs) about the facilitators. " (# CRC 8).*

"There is something that I find that is a great facilitator and it's not always done, it is when the researcher includes knowledge transfer funds in their application for grants……. When it turns in the positive, …., that's a big facilitator." (≠ CRC 4).

The external organisational factors are related to the setting outside the organisation or the clinical environment. Among the external enablers to KT, CRCs expressed the structure of the large research centre (i.e. with its five health regions offering services to the people with visual, hearing, language and mobility disabilities, with various clinicians in rehabilitation and more than fifty researchers in the rehabilitation field) where the proximity to the researchers facilitates networking and collaboration. In addition, there is the opportunity of the networking between CRCs from these health regions. For instance, one CRC says:

"Another positive point that I wanted to bring was the proximity of some researchers physically on partner health regions. This is a great advantage. To be known and all that for the transfer of knowledge, people have the desire to go and hear about [the researcher] because they see her, they know that she is on the partner health region. I think that there is an advantage in that people really take the time to come to the partner health region, that their students come, I think that this is a lever for knowledge transfer." (# CRC 4).

"Our re-networking through large research centre to say that we have access to researchers and knowledge that is collected in other centers, it's a multiplier then…" (# CRC 7).

## Discussion

The aim of this study was to explore CRCs’ scope of practice in KT, the barriers and enablers to their involvement in KT activities, and their needs regarding their knowledge brokering in rehabilitation settings. However, our main observation from the data is that research and KT are often interrelated, and in these rehabilitation centers, part of a larger effort to improve rehabilitation practices. The CRCs talked about their involvement in the research activities before describing their role in KT. Given that KT is a dynamic process, we found that KT activities are a core component of CRCs’ research work. Therefore, these two roles were embedded in their daily activities and were not easily distinguished. The triangulation of data from qualitative and quantitative have been useful to understand better the broad CRCs’ roles in KT.

CRCs in rehabilitation are individuals with diverse backgrounds in both social and biomedical sciences. They serve as a bridge between decision-makers, clinicians and patients/families, and researchers. Given that the research and clinical environments have historically had separate practices and perspectives [27], CRCs face the challenge of finding a common language for the adoption of evidence-informed clinical practices and policies [32]. To bring together divergent perspectives and fulfill their broad role in KT, CRCs expressed the importance of a combination of a core skill such as interpersonal, communication networking, and professional skills. These skills can be mobilized to help ensure their credibility as change agents and increase their self-confidence and the value seen from their work. CRCs are agile in making quick decisions in meetings with stakeholders and are enthusiastic about their marketing role and selling their ideas [59]. According to Ward et al. [30], the interpersonal skills and personal attributes such as flexibility, curiosity and self-confidence are the keys to successful knowledge brokering.

The CRCs in this study viewed KT activities as an integral part of their role. KT is a process that starts during the evaluation of the feasibility and institutional suitability process. This requires a collaborative effort involving researchers, clinical managers, clinicians, and at times service users. According to the CRCs, their KT activities focus on the diffusion, exchange, and spread of knowledge with fewer activities devoted to practice change. Their KT-related activities include organizing conferences, meetings, and seminars, supporting linkages between researchers and knowledge users (e.g. decision-makers and clinicians), and building networks [31, 32, 57, 60]. Some activities match the KB common practices including the knowledge dissemination, sharing and linkage/networking [60].

We found variations in the reported CRCs’ roles and responsibilities. CRCs responsibilities resemble those of their peers in the biomedical area such as the coordination of the research projects, ethical concerns, leadership, data management [38, 61, 62], and the linkage between the researcher and other stakeholders including patients, health professionals, regulatory bodies, and sponsors [35]. However, CRCs in the biomedical field are not involved in the research process following the implementation of scientific evidence in the practice [6]. CRCs in this study are highly qualified personnel hired by the health regions with the expressed purpose to promote the interaction between clinical teams from the health regions and researchers. They work with all researchers in a large rehabilitation research centre, and their roles go beyond those of CRCs in the biomedical field, encompassing some roles and responsibilities consistent with KBs models [30, 31, 63]. From these KBs models, knowledge broking activities can be performed by five role domains including information manager, capacity builder, evaluator, facilitator and linking agent. According to a realist review on the KBs’ role in helping to close the know-do gap [32], KBs and CRCs share several cluster activities reported in existing literature and KB theory such as: 1) an information manager, someone who performs research activities and develops resources based on the needs of those in the local context; 2) a facilitator who reaches out to relevant stakeholders using different means and support the co-learning for stakeholders; or 3) linking agent role between various stakeholders. The majority of KBs in the Canadian rehabilitation context are expert clinicians who tend to perform brokering activities as linking agents, capacity builders, and information roles targeting their peers [33]. Although the relevance of the other KBs’ role domains as a capacity builder referring to skills development to appraise and to apply evidence or as an evaluator associated with the monitoring of clinical practices over time [63], CRCs in this study are not commonly involved in them. However, some of CRCs’ roles could be attributed to the capacity builder as stakeholders learn respectively from each of them and they enable communication through the development of a common language. As an evaluator, CRCs in this study assess the local context to inform their activities. From CRCs, some terminologies refer to their tasks and roles such as gatekeepers, mediators, intermediaries or brokers and facilitators in building the trust relationships within their organizations and outside of them. They are involved in many diffusion and dissemination activities such as knowledge gathering, exchange and sharing. As gatekeeper, CRCs believe that researchers perceive them as a barrier to access clinicians within the organization, referring to their perception as filtering’ roles or regulators of the information. They are points of contact for researchers outside of their organization, and in these roles, they link the organization with its environment and, internally, they play liaison and coordination roles between the research director and the clinical programs’ managers. CRCs reported their roles in the coordination of projects, diffusion and dissemination of knowledge, synthesizing the information and adapting or tailoring for the target population, which aligned with the roles and activities of a KB knowledge manager [23, 30]. As a facilitator and linkage agent [25, 64], CRCs will develop a space for the collaboration, communication and co-creation of learning where researchers and clinical teams will build and maintain a trusting relationship. They will also foster networking through mutual understanding of their needs and expectations. As linkage agent, CRCs will reinforce a research culture among clinical teams (clinicians and managers) in bringing the clinic closer to research. They meet the clinical teams to understand their needs; raise awareness on the benefits of the research, encourage them in the participation of the projects and its co-design; and ultimately to update them on the projects in the respective clinical programs.

CRCs in the current study appear to take similar roles to what Cvitanovic et al. [65] called boundary spanners. Like KBs, boundary spanners have the credibility and facilitate communication and knowledge exchange among many stakeholders. However, boundary spanners represent both sides of the boundary (i.e. the research and clinical settings) unlike KBs who for the most part, are embedded within research teams of organizations [65]. This positioning as the link between research and clinical practice appears to serve both as an obstacle (e.g., confusion about CRCs role) and a facilitator (e.g., strong networking) in the integration of EIP. According to Kislov [27], CRCs have a similar role to hybrid professionals, who use their in-between positions as employees of health regions in the same manner that clinicians and managers are, but external to the clinical programs and working closely to clinical teams and the researchers within the CRIR to support knowledge exchange across within and outside the health regions. CRCs’ roles are defined by their managers in the health regions, and they are accountable to them. CRCs expressed the importance of developing a research culture and building strong relationships between stakeholders by facilitating mutual understanding of each group’s needs and expectations. Despite a greater push from granting agencies and researchers to include integrated KT approaches, the KT process is time-consuming [30] and resource-demanding for both researchers and clinical teams. In such a context, it falls upon the CRCs to fill that gap.

A key finding from this study was the fragmented vision of the CRCs’ role in KT in terms of helping to design research projects and disseminating results. Despite existing research on roles and processes deployed by institutions to promote CRCs continuous involvement in clinical research [37–39, 61, 66], the existing job descriptions for many CRCs and their roles were not clearer for their stakeholders (i.e., the clinical managers, students, clinicians). This highlighted the importance of strong communication to improve not only their roles but also to increase the “marketing of their roles”. Furthermore, there is no standard job description, and no agreement on what the CRC role entails or what skills/qualifications are required [31, 67]. As a result of not having a shared vision, KT activities are often unstandardized, hindering the successful implementation of this role as well as the recognition of CRCs’ effectiveness in the uptake of EIP. Unfortunately, this lack of clarity about the CRC’s role is believed to negatively influence their potential to affect KT processes and outcomes.

CRCs noted several factors influencing their work, more related to organizational factors at the meso level (e.g. organizational culture, climate, structures, and support) or between the micro and meso levels (e.g. financial resources, leadership, time availability) [58]. Participants mentioned that the receptivity and readiness of the organization were vital for the success of their KT activities. In fact, the organizational context (e.g. readiness for change, organizational research culture, organizational climate and culture, readiness, time availability, financial resources [56]) is frequently reported as a key enabler in the KT literature [8, 54, 56, 58, 68]. However, their position referring to hybrid professionals and the confusion related to their roles are the barriers to their KT activities. According to the literature on the context, CRCs seem to have a more dynamic vision of the context meaning that they can rely on the support from their managers and that of their colleagues to improve their roles [58]. A notable facilitator identified through this study is the strong inter-health regions networking of CRCs where participants reported that this model is conducive to knowledge sharing and exchange. From Ward et al. [30], a better understanding of contextual factors influencing KBs is needed to support and justify the commitment to resources to them. Managerial support of research and the proximity with the clinical team, as well the quick access to the researchers may facilitate the CRC’s work in KT activities. This is consistent with the findings regarding KBs’ commitment to the implementation of an evidence-based practice and in optimizing impact on clinical practice [69]. Factors like time and the availability of resources are both barriers and facilitators. According to Ward et al. [30], KB is time-consuming regardless of the type of model that is used ( i.e. knowledge management, linkage and exchange or capacity development models). Indeed, KB requires time and resources for identifying and sharing research results, building relationships, and creating partnerships, and capacity building.

Finally, CRCs identified their needs with respect to KT and suggested strategies to mitigate these challenges and to support them in their role. Although CRCs agree or strongly agree that they are confident, valued, autonomous and competent in their work, the majority would like to have support or training to fulfil their roles. For instance, they suggested that digital solutions such social media, and videoconferences could help with efficiency. The need for additional skills and competencies (e.g. project management, social medias) was also voiced by them [26].

Boutcher et al. [69, 70] and Urquhart et al. [69, 70] have found that opportunities for professional development may help build the competencies that are required to encourage a substantial improvement in the reduction of the gaps in health care and evidence-informed decision-making [69, 70]. Using a marketing approach was suggested as a promising avenue to reinforce their message and to raise stakeholder awareness of their roles and responsibilities. CRCs perceive that they would be more active in KT activities if a clear action plan with KT indicators, strategies, and processes was consistently available. An action plan could indeed be the missing link and opportunity to formally recognize their roles, but also to ensure that KT activities are aligned with the local needs of the organizations.

### Strengths and limitations

The use of a participatory approach and mixed methods provided a comprehensive picture of CRCs’ roles in the research and KT, as well as the factors influencing these roles. For example, the diversity of CRCs from different partner health regions in the same research centre, different data collection methods, and peer review of the preliminary analysis increased the triangulation of diverse perspectives. However, the small sample size and the local context of rehabilitation, can influence the generalisability of these results. While this exploratory study was to examine the role and the contributions of CRCs in KT, we found KT activities are a component of CRCs’ research work. Therefore, these two roles were embedded in their daily activities and were not easily distinguished. All CRCs adhere to an iterative approach to knowledge transfer whenever possible, hence their shared vision of integrating KT into all aspects of research, from the first idea for a project to its completion. Through their roles, CRCs are similar to other roles in the literature such KBs [30, 31, 33, 63]. Then, we believe that this exploratory study contributes to advancing knowledge on the topic. In addition, the findings from the two data collection approaches captured CRCs’ roles in research and KT as well as the needed support they would like to have to optimise their role. Another limitation was the lack of data collection on the CRCs’ job descriptions. Although this was beyond the scope of this study, further exploration of job descriptions and comparison with the participants’ perception of their roles could potentially assist in developing strategies to reinforce CRCs’ roles among their stakeholders. CRCs did mention the commonalities between their roles and the opportunities of networking during the data collection.

 An interesting area for the future research would have been to explore CRCs’ perceptions of the extent to which knowledge brokering strategies can help achieve changes in practice and policy.

## Conclusion

This study provides valuable insights into rehabilitation CRCs’ activities and scope of work. The results highlighted the complexity of CRCs’ role and profiles, which were consistent with the existing literature on CRCs in the biomedical field and KBs in rehabilitation and healthcare more broadly. CRCs promote collaborations between researchers and knowledge users by providing the latter with access to research knowledge that can improve their clinical practices. Key factors influencing their activities include contextual variables related to organizational culture and climate. A shared vision of KT, and alignment of expectations between the large rehabilitation research centre and partner health region managers, as well as marketing to promote this role were suggested to overcome barriers and maximally fulfill their role in promoting EIP. The data synthesis of CRCs’ roles, activities, factors, and strategies/needs led us to develop a matrix of our findings (attributes, roles, activities, strategies, enablers, barriers) (Additional file 1) in an organizing framework to capture our interpretation of how CRCs function as KBs and bounder spanners in healthcare organizations. Beyond the practice of rehabilitation, our findings can be applied to a wide range of individuals and organizations who are expected to be involved in knowledge brokering to improve EIP. The insights gained from this study can potentially inform other collaborative approaches in knowledge networks and other boundary organizations involved in KT activities with multiple stakeholders.

## Supplementary Information

**Additional file 1.** Matrix of CRCs in the rehabilitation area.

## Data Availability

The datasets used and/or analysed can be made available from the corresponding author upon request.
