# Supplementary material for: Clinical research coordinators’ role in knowledge translation activities in rehabilitation: a mixed methods study
Source: BMC Health Serv Res. 2023 Feb 7;23:124. doi: 10.1186/s12913-023-09027-0 (PMC9903418; doi:10.1186/s12913-023-09027-0)
Supplement: Supplementary file 1 — Additional file 1. Matrix of CRCs in the rehabilitation area. [file 12913_2023_9027_MOESM1_ESM.docx]

**Additional file** **1: Matrix of CRCs in the rehabilitation area**

**Attributes/Characteristics**

**Self-confidence**

**Valued**

**Respected**

**Autonomous**

**Enjoyed**

**Credibility**

**Flexibility, agility, humility listener**

**Communicator,**

**Facilitator**

**Synthesis skills**

**Strategic skills**

**Management skills**

**Roles**

**Information manager**

**Facilitator**

**Linkage agent**

**Other: bridge, brokers, bound spanner, gatekeeper, capacity builder, transmission belt, mediator, intermediary**

**Facilitation**

**Time;**

**Technological and human resources;**

**Have training**

**Budget**

**Alignment & marketing of CRCs’ role;**

**Clear action plan with the targets and expectations**

**Influencing/Contextual factors**

**Lack of time**

**Lack of financial, human and technological resources**

**Confusion about CRCs’ roles**

**Managers’ duality between the clinical expectations and the commitment in research**

**Cultural difference between the knowledge producers and the knowledge users**

**Supportive local environment**

**Characteristics of the large research centre**

**KT activities**

**Knowledge diffusion and dissemination**

**Assessing, synthetizing and tailoring**

**Coordination**

**Communication**

**Linkage between two worlds with differents language and culture**
